# Cardiovascular burden and unemployment: A retrospective study in a large population-based French cohort

**DOI:** 10.1371/journal.pone.0288747

**Published:** 2023-07-17

**Authors:** Marina Sanchez Rico, Marie Plessz, Guillaume Airagnes, Céline Ribet, Nicolas Hoertel, Marcel Goldberg, Marie Zins, Pierre Meneton

**Affiliations:** 1 AP-HP, DMU Psychiatrie et Addictologie, Hôpital Corentin-Celton, Issy-les-Moulineaux, France; 2 Centre Maurice Halbwachs, INRAE, EHESS, ENS-PSL, CNRS, Paris, France; 3 AP-HP, DMU Psychiatrie et Addictologie, Hôpital Européen Georges-Pompidou, Paris, France; 4 Université Paris Cité, Faculté de Médecine, Paris, France; 5 UMS_011, INSERM, Université Paris-Saclay, Villejuif, France; 6 UMR_1266, INSERM, Paris, France; 7 UMR_1142, INSERM, Sorbonne Université, Paris, France; Shahid Beheshti University of Medical Sciences School of Medicine, ISLAMIC REPUBLIC OF IRAN

## Abstract

The specific effect of unemployment on cardiovascular health relatively to the effects of social position and work environment is still unclear. To clarify this effect, the associations between current or past unemployment and the prevalence of common cardiovascular risk factor and events were tested using multiple logistic regression models with adjustment for both social position and prior work environment. The analyses were performed in a population-based French cohort (CONSTANCES) that included 131,186 adults enrolled between 2012 and 2021. Participants who were unemployed at inclusion (n = 8278) were overexposed to non-moderate alcohol consumption, smoking, leisure-time physical inactivity and depression (odds ratios (ORs) from 1.19 to 1.58) whereas those who have been unemployed at least once in the past (n = 19,015) were additionally overexposed not only to the previous risk factors but also to obesity, diabetes and sleep disorders (ORs from 1.10 to 1.35). These latter were also more exposed to non-fatal myocardial infarction and peripheral arterial disease (ORs of 1.44 and 1.47 respectively), overexposures that persisted after further adjustment for cardiovascular risk factors (ORs of 1.36 and 1.33). The overexposures to risk factors and cardiovascular events were both dependent on the duration of past unemployment. They were equally observed in participants with low social position or bad work environment. These results suggest that unemployment increases cardiovascular risk independently from social position and work environment with a cumulative effect over time. The effect of unemployment could add up to those of low social position and bad work environment during lifetime to further increase cardiovascular risk. They also suggest that long-term unemployment increases the prevalence of cardiovascular events through pathways including but not limited to overexposure to common risk factors.

## Introduction

Unemployment is associated with a high incidence of cardiovascular diseases [1–6], possibly translating into premature mortality [7]. The mechanisms by which unemployment would cause cardiovascular problems remain elusive although overexposure to behavioral risk factors such as alcohol consumption, smoking, unbalanced diet or low physical activity is likely involved [8].

Disentangling the specific effect of unemployment is difficult because it is strongly interrelated with social position which is a powerful determinant of cardiovascular health [9]. For example, individuals with low social position, as measured by educational level, occupational class or income, have a higher risk of coronary heart disease [10] partly due to overexposure to several risk factors, including smoking, alcohol consumption, leisure-time physical inactivity, obesity, diabetes, hypertension, dyslipidemia, depression and sleep disorders [11–17]. Whatever the reasons why individuals with low social position more often adopt unhealthy behaviors and have higher cardiovascular risk, which comprise material deprivation, educational and cultural attainment, the importance given to the care of one’s own health, the ability to cope with illness and to access health care [18], they might explain part or all of the observed association between unemployment and high cardiovascular risk. Alternatively, individuals with low social position who consequently have high cardiovascular risk could be proportionally less affected by unemployment than socially privileged individuals whose cardiovascular risk is low.

Unemployment is also strongly interrelated with work environment which is another powerful determinant of cardiovascular health [19]. Individuals with bad working conditions are overexposed to alcohol consumption, smoking, leisure-time physical inactivity, obesity, hypertension, diabetes, sleep disorders and depression [20–27] and accordingly have an increased risk of coronary heart disease [28]. Bad work environment, which is interrelated with low social position [29], may also explain part or all of the association between unemployment and high cardiovascular risk. Another possibility is that individuals with bad work environment, who as a consequence have high cardiovascular risk, might be proportionally less sensitive to the effect of unemployment compared to individuals with good work environment, job exit appearing somewhat like a salvation.

In order to sort out the specific contribution of unemployment, the present study took advantage of a large population-based cohort to assess the association between past and/or current unemployment and the prevalence of common cardiovascular risk factors and events, while taking into account both social position and work environment. The results strongly suggest that unemployment has an independent effect on cardiovascular health which could add up to those of social position and work environment. They also suggest that long-term unemployment augments the risk of cardiovascular events not only by increasing the exposure to common behavioral and clinical risk factors but also through other pathways that remain to be defined.

## Methods

### Study population

205,203 adults who were affiliated to the general health insurance system (which covers 85% of the French population) were enrolled in the CONSTANCES cohort between February 2012 and September 2021 using a random sampling scheme stratified on age, sex, socioeconomic status and region [30]. Inclusion criteria comprised the obligation to provide written informed consent, to undergo a comprehensive health examination in one of the twenty-one participating medical centers scattered across metropolitan territory and to complete self-administered questionnaires on lifestyle, health-related behaviors, social and occupational conditions. The inclusion rate was rather low (7.3%) [31] in line with those observed in other large population-based cohorts when participants are required to visit a medical center for health-related exams [32]. Note that the authors of the present study did not have access to information that could have identified individual participants during or after data collection. The cohort received approvals from the Ethics Evaluation Committee of the French National Institute of Health and Medical Research and from the National Committee for the Protection of Privacy and Civil Liberties.

The analyses were performed in a subset of 131,186 participants who had no missing values in all variables that were included in multi-adjusted regression models. The percentages of excluded participants for each cardiovascular risk factor and the potential differences in these percentages between levels of each risk factor suggest that missing data are not randomly distributed (S1 Table). The choice of selecting participants with no missing values rather than imputing non-random missing data, which would not have been devoid of biases [33], was driven by the fact that the cohort was not representative of the French population due to the low inclusion rate and the resulting selection of socially privileged people and that the selection of participants with no missing values only marginally accentuated this bias (S2 Table).

### Social position of participants

Several socioeconomic indicators whose distributions are shown in S3 Table were considered for assessing social position of participants at inclusion. Educational attainment was classified into four levels depending on the number of years of study: ≤11, 12–13, 14–16 or ≥17. Occupation of participants and spouses was reduced from a ten-level classification in the original inquiry to three grades: blue collar/clerk, intermediate and management. Income that included monthly earnings of all household members was ranked as low (below 1500 euros), middle (between 1500 and 2800 euros), high (between 2800 and 4200 euros) or very high (above 4200 euros). These thresholds were dictated by the inquiry that originally included seven levels of income and the need to balance the number of participants between groups. Social vulnerability was evaluated by a score calculated from a questionnaire comprising 11 binary items (Y/N) exploring material and social deprivation [34]: “do you sometimes meet a social worker?”, “do you have complementary health insurance?”, “do you live as a couple?”, “are you a homeowner?”, “are there periods in the month when you have real financial difficulties to meet your basic needs?”, “have you done any sports activities in the last 12 months?”, “have you been to any show over the last 12 months?”, “have you been on holiday over the last 12 months?”, “have you seen any family member over the last six months?”, “if you have difficulties, is there anyone around who could take you in for a few days?”, “if you have difficulties, is there anyone around who could provide you with material assistance?”. This score was categorized into terciles (low, intermediate or high social vulnerability) for the analyses. Note that participants who were unemployed at inclusion reported the occupation, income and social vulnerability status they had just before the unemployment episode.

Given that these different indicators assess complementary and interdependent aspects of social position (S1 Fig), a global score was calculated by giving for each indicator a value of 1 to the least privileged group, 2 or 3 to intermediary groups and 3 or 4 to the most privileged group, depending if the indicator encompassed 3 or 4 levels, by summing the values and by dividing the sum by the number of available indicators for each participant. This global score was categorized into terciles (low, middle or high social position) for the analyses, as previously reported [35].

### Work environment of participants

A total of 19 occupational exposures whose distributions are shown in S4 Table were used to characterize work environment of participants at inclusion. These included a series of organizational, physical, biomechanical, chemical and psychosocial factors such as commuting time, clocking in and out, regular working hours (on daily and weekly basis), long working hours (over 10h per week day), night work, dealing with the public, driving on public road, repetitive work (imposed by a machine, a procedure or someone), working with a screen, standing work posture, handling heavy loads (over one kilogram), physically demanding work, exposure to vibrations, exposure to noise, outdoor work, working in the cold, working in the heat, exposure to chemicals and the scale assessing effort-reward imbalance of work that was divided into terciles (low, average or high imbalance) [36]. Note that participants who were unemployed at inclusion reported the work environment they had just before becoming unemployed.

Work environment was considered as a whole, which is reality for workers who are not facing only one or a few occupational exposures [37]. For that purpose, the exposures that were significantly interrelated with each other (S2 Fig) were combined into a global score that was calculated by giving for each exposure a value of 1 to the least exposed group, 3 to the more exposed group, and 2 to intermediary groups whenever the exposure encompassed 3 levels, by summing the values and by dividing the sum by the number of available exposures for each worker. This global score was categorized into terciles (bad, average or good work environment) for the analyses, as already described [38].

### Unemployment experienced by participants

The employment status of participants at inclusion was primarily assessed by a question with multiple choices, allowing to describe complex situations. Possible answers were: “I have a job (even if on sick leave, unpaid leave or availability, maternity, paternity, adoption or parental leave)”, “unemployed or job seeker”, “retired or no longer in business”, “in training (pupil, student, trainee, apprentice, etc.)”, “I do not work for health reasons (long-term illness, disability)”, “no professional activity”. Participants who checked the box “I have a job”, and only this one, were considered employed. Participants who checked the box “unemployed or job seeker” were considered unemployed only if they had not also checked the boxes “I have a job” or “I do not work for health reasons”. Several other questionnaires, which asked more briefly whether participants held a job, allowed to confirm their employment status at inclusion. Past unemployment was documented by a specific questionnaire in which participants were asked to report each time they had stopped working for a period of more than six months and why (unemployment, health issue, other reason). The existence of past unemployment was confirmed by administrative data from the French national pension system that additionally provided the number of unemployed quarters and the year they occurred during lifetime for each participant. The number of unemployed quarters was categorized into terciles and used to measure the duration of past unemployment. Unemployment at inclusion (Y/N) and unemployment at least once in the past (Y/N), whatever the number of unemployed quarters, were considered as two separate variables for the analyses. In particular, the duration of the episode of unemployment at inclusion, which was not available, was not included in the number of unemployed quarters.

### Prevalence of cardiovascular risk factors and events among participants

Several risk factors which are commonly found in the population were considered. These included three nonmodifiable factors: sex, age that was divided into terciles and parental history of cardiovascular event that was coded as a binary variable (Y/N) referring to the occurrence of an event on father’s or mother’s side, whatever their age. Three behavioral factors: smoking coded into three categories (currently, former, never), lifetime non-moderate alcohol consumption (more than two or three drinks on the same day in women or men, respectively) [39] classified as rarely (never or less than 1 time per month), sometimes (two or three times per month) or often (one time or more per week) and leisure-time physical inactivity whose inquiry was based on a three item questionnaire asking about regular practice of walking or cycling, practicing a sport and gardening or housekeeping over the past 12 months; each item was noted 0 if the answer was no, 1 if the practice was regular but low (less than 15 minutes for sport, or 2 hours for the two other items, per week), 2 if the practice was regular and higher; the score calculated by summing the three items ranged from 0 (not active at all) to 6 (very active) and was used to characterize leisure-time physical inactivity (participants with a score <2). Six clinical risk factors were retained: body mass index, hypertension, dyslipidemia (either hypercholesterolemia or hypertriglyceridemia), diabetes, sleep disorders and depression. The inquiry into the presence and the age of onset of hypertension, dyslipidemia, diabetes and sleep disorders, which were coded as binary variables (Y/N), was performed by physicians in the medical centers. Body mass index (BMI) was calculated from measured weight and height and coded into three categories (optimal if BMI <25 kg/m^2^, overweight if 25≤ BMI <30 kg/m^2^, obesity if BMI ≥30 kg/m^2^). Depression was assessed using the Centre of Epidemiologic Studies Depression scale and defined as a score ≥19 rather than ≥16 in both sexes [40].

Behavioral and clinical risk factors as defined above were used for adjustment purpose in the regression models calculating odds ratios for cardiovascular events. They were also used as dependent variables in the models calculating their own odds ratios; in this case, smoking, non-moderate alcohol consumption (more than two or three drinks on the same day in women or men, respectively) and obesity (BMI ≥30 kg/m^2^) at inclusion were coded as binary variables (Y/N). Non-moderate alcohol consumption referring to drinking habits during the week before completing the questionnaire.

Physicians also inquired about any non-fatal cardiovascular event and the age at which it occurred during the lifetime of participants. Four types of events, coded as binary variables (Y/N), were considered for the analyses: stroke, myocardial infarction, angina pectoris and peripheral arterial disease.

As the validity of self-reported information, even when collected by physicians, can be questioned, the coherence of the relationships between risk factors and cardiovascular events was tested (S5 Table). The fact that most of the expected associations were observed was a good indication that the collected information was reliable. In any case, if a bias was present, it would likely have been under-reporting with rates varying from one event to another: 77.8% for peripheral arterial disease, 72.4% for myocardial infarction, 71.4% for angina pectoris, 54.5% for stroke [41].

### Statistical analyses

The characteristics of cohort participants with or without missing values or of individuals randomly selected from the French population were compared by pairs using Cohen’s h measure of effect size with the rule of thumb to categorize substantial differences as small (0.2 ≤ h < 0.5), medium (0.5 ≤ h > 0.8) or large (h ≥ 0.8) [42].

The characteristics of participants according to their past or current experience of unemployment were compared by calculating standardized mean differences (SMD); values above 0.1 being considered as showing significant differences [43]. SMD was computed using the R package ‘tableone’ 0.13.2 [44] with R software 4.2.2.

The associations between the prevalence of cardiovascular risk factors at inclusion and past or current unemployment were tested with multiple logistic regression modeling. Two types of models were used: models 1 were adjusted for sex and age; models 2 were adjusted for sex, age, social position, work environment and current or past unemployment. The associations between the prevalence of non-fatal cardiovascular events at inclusion and past or current unemployment were tested by using three types of models: models 1 were adjusted for sex, age and parental history of cardiovascular event; models 2 were adjusted for sex, age, parental history of cardiovascular event, social position, work environment and past or current unemployment; models 3 were adjusted for sex, age, parental history of cardiovascular event, social position, work environment, past or current unemployment, lifetime alcohol consumption, smoking, leisure-time physical inactivity, body mass index, hypertension, dyslipidemia, diabetes, sleep disorders and depression.

Residual analyses were performed to assess the fit of the data, assumptions were checked and the potential influence of outliers was examined for all associations [45]. Statistical significance was fixed a priori at two-sided p-value <0.05. All analyses were performed with the statistical discovery software JMP 17 Pro (SAS, Cary NC).

## Results

### Association between social position, work environment and past or current unemployment of participants

As shown in S3 Fig, social position and work environment of participants were highly correlated, the lower the social position, the worse the work environment (SMD = 0.665). Compared to participants who never encountered unemployment, those who have been unemployed at least once had a lower social position and worse work environment (Table 1). More specifically, they were less educated, they and their spouse were more often blue collars or clerks, they had a lower income and were socially more vulnerable (S3 Table). Several indicators used to characterize their work environment were worse, particularly repetitive work, handling heavy loads, physically demanding work, exposure to noise and exposure to chemicals (S4 Table). Participants who were unemployed at inclusion had also a lower social position and worse work environment and experienced much more frequently unemployment in the past (SMD = 0.775) than those who were employed (S6 Table).

**Table 1:** Cardiovascular risk factors in participants at inclusion according to their past experience of unemployment.

		Past unemployment				SMD
		Never (n = 112,171)		At least once (n = 19,015)		
		n	%	n	%	
**Sex**	**Women**	56,476	84.0	10,767	16.0	0.126
	**Men**	55,695	87.1	8248	12.9	
**Age (y)**	**18–39**	37,626	88.8	4743	11.2	0.195
	**40–54**	37,103	83.2	7466	16.8	
	**55–75**	37,442	84.6	6806	15.4	
**Parental history of cardiovascular event**	**No**	85,545	85.9	14,038	14.1	0.056
	**Yes**	26,626	84.3	4977	15.7	
**Current unemployment**	**No**	107,631	87.6	15,277	12.4	0.498
	**Yes**	4540	54.8	3738	45.2	
**Social position**	**High**	34,526	90.4	3685	9.64	0.325
	**Middle**	53,668	79.2	9027	20.8	
	**Low**	23,977	85.6	6303	14.4	
**Work environment**	**Good**	36,015	87.3	5224	12.7	0.129
	**Average**	40,114	83.4	6615	16.6	
	**Bad**	36,042	85.8	7176	14.2	
**Lifetime non-moderate** **alcohol consumption**	**Rarely**	15,415	83.2	3106	16.8	0.078
	**Sometimes**	23,343	85.2	4044	14.8	
	**Often**	73,413	86.1	11,865	13.9	
**Smoking**	**Never**	53,371	88.1	7181	11.9	0.204
	**Former**	38,113	83.9	7334	16.1	
	**Current**	20,687	82.1	4500	17.9	
**Leisure-time physical inactivity**	**No**	102,089	85.7	17,056	14.3	0.045
	**Yes**	10,082	83.7	1959	16.3	
**Body mass index**	**Optimal**	66,700	86.4	10,484	13.6	0.120
	**Overweight**	33,569	85.3	5808	14.7	
	**Obese**	11,902	81.4	2723	18.6	
**Hypertension**	**No**	101,331	85.7	16,963	14.3	0.037
	**Yes**	10,840	84.1	2052	15.9	
**Dyslipidemia**	**No**	104,279	85.5	17,621	14.5	0.011
	**Yes**	7892	85.0	1394	15.0	
**Diabetes**	**No**	110,586	85.6	18,650	14.4	0.040
	**Yes**	1585	81.3	365	18.7	
**Sleep disorders**	**No**	41,870	87.0	6257	13.0	0.093
	**Yes**	70,301	84.6	12,758	15.4	
**Depression**	**No**	97,332	86.5	15,147	13.5	0.191
	**Yes**	14,839	79.3	3868	20.7	

The percentages were calculated relatively to the number of participants in each risk factor level; the differences between past unemployment experiences were assessed by computing standardized mean differences (SMD).

### Characteristics and cardiovascular burden of participants at inclusion according to their past experience of unemployment

Compared to participants who never faced unemployment, those who were unemployed at least once in the past (19,015 participants representing 14.5% of the cohort) were more likely to be middle-aged women with low social position, bad work environment and unemployed at inclusion (Table 1). They were overexposed to several cardiovascular risk factors, including non-moderate alcohol consumption, smoking, leisure-time physical inactivity, obesity, diabetes, sleep disorders and depression, with odds ratios ranging from 1.10 (non-moderate alcohol consumption) to 1.35 (depression) after adjustment for sex, age, social position, work environment and current unemployment (Table 2). The overexposure to risk factors was associated with an increased prevalence of non-fatal myocardial infarction and peripheral arterial disease with odds ratios of 1.36 and 1.33 respectively after adjustment for sex, age, parental history of cardiovascular event, social position, work environment, current unemployment and risk factors (Table 3).

**Table 2:** Adjusted odds ratios (95% confidence interval) for the prevalence of cardiovascular risk factors in participants at inclusion according to their past experience of unemployment.

	Past unemployment	n	%	Models 1	p	Models 2	p
**Non-moderate alcohol consumption**	**Never**	11,606	10.3	1.00		1.00	
	**At least once**	2217	11.7	1.20 (1.14–1.26)	<0.0001	1.10 (1.04–1.15)	0.0003
**Smoking**	**Never**	20,687	18.4	1.00		1.00	
	**At least once**	4500	23.7	1.47 (1.42–1.53)	<0.0001	1.24 (1.19–1.29)	<0.0001
**Leisure-time physical inactivity**	**Never**	10,082	9.0	1.00		1.00	
	**At least once**	1959	10.3	1.19 (1.13–1.26)	<0.0001	1.12 (1.06–1.18)	<0.0001
**Obesity**	**Never**	11,902	10.6	1.00		1.00	
	**At least once**	2723	14.3	1.36 (1.30–1.42)	<0.0001	1.20 (1.14–1.25)	<0.0001
**Hypertension**	**Never**	10,840	10.0	1.00		1.00	
	**At least once**	2052	10.8	1.08 (1.02–1.14)	0.005	1.05 (0.99–1.11)	0.08
**Dyslipidemia**	**Never**	7892	7.0	1.00		1.00	
	**At least once**	1394	7.3	1.02 (0.95–1.08)	0.63	0.99 (0.93–1.06)	0.87
**Diabetes**	**Never**	1585	1.4	1.00		1.00	
	**At least once**	365	1.9	1.37 (1.22–1.54)	<0.0001	1.24 (1.10–1.40)	0.0006
**Sleep disorders**	**Never**	70,301	62.7	1.00		1.00	
	**At least once**	12,758	67.1	1.20 (1.16–1.24)	<0.0001	1.15 (1.11–1.19)	<0.0001
**Depression**	**Never**	14,839	13.2	1.00		1.00	
	**At least once**	3868	20.3	1.64 (1.57–1.70)	<0.0001	1.35 (1.30–1.41)	<0.0001

The percentages were calculated relatively to the number of participants in each past experience of unemployment (never = 112,171; at least once = 19,015).

Models 1 were adjusted for sex and age.

Models 2 were adjusted for sex, age, current unemployment, social position and work environment.

**Table 3:** Adjusted odds ratios (95% confidence interval) for the prevalence of non-fatal cardiovascular events in participants at inclusion according to their past experience of unemployment.

	Past unemployment	n	%	Models 1	p	Models 2	p	Models 3	p
**Stroke**	**Never**	777	0.69	1.00		1.00		1.00	
	**At least once**	133	0.70	0.96 (0.80–1.15)	0.66	0.91 (0.75–1.11)	0.35	0.90 (0.74–1.09)	0.28
**Myocardial infraction**	**Never**	673	0.60	1.00		1.00		1.00	
	**At least once**	157	0.83	1.47 (1.24–1.76)	<0.0001	1.44 (1.20–1.73)	<0.0001	1.36 (1.12–1.64)	0.001
**Angina pectoris**	**Never**	612	0.55	1.00		1.00		1.00	
	**At least once**	123	0.65	1.24 (1.02–1.51)	0.03	1.22 (0.99–1.49)	0.06	1.18 (0.96–1.45)	0.12
**Peripheral arterial disease**	**Never**	207	0.18	1.00		1.00		1.00	
	**At least once**	55	0.29	1.63 (1.21–2.20)	0.001	1.47 (1.08–2.01)	0.01	1.33 (1.01–1.82)	0.04

The percentages were calculated relatively to the number of participants in each past experience of unemployment (never = 112,171; at least once = 19,015).

Models 1 were adjusted for sex, age and parental history of cardiovascular event.

Models 2 were adjusted for sex, age, parental history of cardiovascular event, current unemployment, social position and work environment.

Models 3 were adjusted for sex, age, parental history of cardiovascular event, current unemployment, social position, work environment, lifetime alcohol consumption, smoking, leisure-time physical inactivity, obesity, hypertension, dyslipidemia, diabetes, sleep disorders and depression.

### Cardiovascular burden of participants at inclusion according to the duration of their past experience of unemployment

The number of quarters during which participants were unemployed in the past was associated with the prevalence of at least three cardiovascular risk factors, namely non-moderate alcohol consumption, smoking and depression (Table 4). The higher the number of quarters, the higher the prevalence of these risk factors, with odds ratios increasing in a dose-dependent manner after adjustment for sex, age, social position, work environment and current unemployment (Table 4). In accordance with the overexposure to risk factors, the prevalence of non-fatal myocardial infarction and peripheral arterial disease was higher only in the group of participants who were unemployed for the longest time, even though the odds ratio did not reach statistical significance in the case of peripheral arterial disease due to the small number of events, after adjustment for sex, age, parental history of cardiovascular event, social position, work environment, current unemployment and risk factors (Table 5).

**Table 4:** Adjusted odds ratios (95% confidence interval) for the prevalence of cardiovascular risk factors in participants at inclusion according to the duration of their past experience of unemployment.

	Past unemployment (quarters, n)	n	%	Models 1	p	Models 2	p
**Non-moderate alcohol consumption**	**1–9**	587	10.2	1.00		1.00	
	**10–20**	815	11.4	1.20 (1.07–1.34)	0.002	1.15 (1.02–1.29)	0.02
	**21–148**	815	13.3	1.45 (1.29–1.63)	<0.0001	1.33 (1.17–1.50)	<0.0001
**Smoking**	**1–9**	1232	21.5	1.00		1.00	
	**10–20**	1651	23.1	1.24 (1.13–1.35)	<0.0001	1.13 (1.04–1.24)	0.005
	**21–148**	1617	26.3	1.89 (1.72–2.07)	<0.0001	1.54 (1.40–1.69)	<0.0001
**Leisure-time physical inactivity**	**1–9**	600	10.5	1.00		1.00	
	**10–20**	754	10.6	1.06 (0.95–1.19)	0.30	1.03 (0.92–1.16)	0.60
	**21–148**	605	9.8	1.10 (0.97–1.25)	0.13	1.02 (0.89–1.16)	0.81
**Obesity**	**1–9**	700	12.2	1.00		1.00	
	**10–20**	992	13.9	1.11 (1.01–1.23)	0.04	1.03 (0.92–1.14)	0.61
	**21–148**	1031	16.8	1.26 (1.13–1.40)	<0.0001	1.05 (0.94–1.17)	0.41
**Diabetes**	**1–9**	85	1.5	1.00		1.00	
	**10–20**	119	1.7	1.05 (0.79–1.39)	0.75	1.00 (0.75–1.33)	0.99
	**21–148**	161	2.6	1.23 (0.94–1.62)	0.13	1.08 (0.81–1.43)	0.61
**Sleep disorders**	**1–9**	3748	65.4	1.00		1.00	
	**10–20**	4808	67.3	1.09 (1.01–1.17)	0.03	1.04 (0.97–1.13)	0.26
	**21–148**	4202	68.4	1.17 (1.08–1.27)	<0.0001	1.08 (0.99–1.17)	0.07
**Depression**	**1–9**	950	16.6	1.00		1.00	
	**10–20**	1443	20.2	1.27 (1.16–1.39)	<0.0001	1.14 (1.03–1.25)	0.007
	**21–148**	1475	24.0	1.68 (1.52–1.84)	<0.0001	1.29 (1.17–1.43)	<0.0001

The percentages were calculated relatively to the number of participants in each tercile of past unemployment quarters (1–9 = 5730; 10–20 = 7141; 21–148 = 6144).

Models 1 were adjusted for sex and age.

Models 2 were adjusted for sex, age, current unemployment, social position and work environment.

**Table 5:** Adjusted odds ratios (95% confidence interval) for the prevalence of non-fatal myocardial infarction and peripheral arterial disease in participants at inclusion according to the duration of their past experience of unemployment.

	Past unemployment (quarters, n)	n	%	Models 1	p	Models 2	p	Models 3	p
**Myocardial infraction**	**1–9**	31	0.54	1.00		1.00		1.00	
	**10–20**	46	0.64	1.15 (0.73–1.83)	0.54	1.16 (0.73–1.84)	0.54	1.15 (0.72–1.86)	0.55
	**21–148**	80	1.30	1.75 (1.15–2.67)	0.009	1.75 (1.13–2.70)	0.01	1.75 (1.12–2.74)	0.01
**Peripheral arterial disease**	**1–9**	9	0.16	1.00		1.00		1.00	
	**10–20**	16	0.22	1.36 (0.60–3.10)	0.46	1.39 (0.61–3.17)	0.43	1.34 (0.58–3.12)	0.49
	**21–148**	30	0.49	2.28 (1.07–4.85)	0.03	2.35 (1.08–5.09)	0.03	2.14 (0.96–4.74)	0.06

The percentages were calculated relatively to the number of participants in each tercile of past unemployment quarters (1–9 = 5730; 10–20 = 7141; 21–148 = 6144).

Models 1 were adjusted for sex, age and parental history of cardiovascular event.

Models 2 were adjusted for sex, age, parental history of cardiovascular event, current unemployment, social position and work environment.

Models 3 were adjusted for sex, age, parental history of cardiovascular event, current unemployment, social position, work environment, lifetime alcohol consumption, smoking, leisure-time physical inactivity, obesity, hypertension, dyslipidemia, diabetes, sleep disorders and depression.

### Characteristics and cardiovascular burden of participants at inclusion according to their current experience of unemployment

Compared to participants who were employed at inclusion, those who were unemployed at that time (8278 participants representing 6.3% of the cohort) were more likely to be young with low social position, bad work environment and past experience of unemployment (S6 Table). They were overexposed to several cardiovascular risk factors, including non-moderate alcohol consumption, smoking, leisure-time physical inactivity and depression, with odds ratios ranging from 1.19 (leisure-time physical inactivity) to 1.58 (depression) after adjustment for sex, age, social position, work environment and past unemployment (S7 Table). However, this overexposure to risk factors did not translate into an increased prevalence of non-fatal cardiovascular events after adjustment for sex, age, parental history of cardiovascular event, social position, work environment, past unemployment and risk factors (S8 Table).

### Cardiovascular burden of participants with low social position or bad work environment according to their experience of unemployment

When considering only participants with low social position (30,280 participants representing 23.1% of the cohort), those who were unemployed at least once in the past compared to those who never encountered unemployment were overexposed to at least four cardiovascular risk factors, namely smoking, obesity, sleep disorders and depression, with odds ratios ranging from 1.13 (obesity) to 1.30 (depression) after adjustment for sex, age, work environment and current unemployment (S9 Table). The overexposure to risk factors was associated with an increased prevalence of non-fatal myocardial infarction with an odds ratio of 1.50 after adjustment for sex, age, parental history of cardiovascular event, work environment, current unemployment and risk factors (S10 Table). Participants with low social position who were unemployed at inclusion compared to those who were employed at that time were overexposed to at least five risk factors, including non-moderate alcohol consumption, smoking, leisure-time physical inactivity, obesity and depression, with odds ratios ranging from 1.18 (leisure-time physical inactivity) to 1.52 (smoking) after adjustment for sex, age, work environment and past unemployment (S11 Table). No significant interaction was found between social position and past or current unemployment on the prevalence of risk factors and cardiovascular events (results not shown).

When considering only participants with bad work environment (43,218 participants representing 32.9% of the cohort), those who were unemployed at least once in the past compared to those who never encountered unemployment were overexposed to at least six cardiovascular risk factors, namely smoking, leisure-time physical inactivity, obesity, diabetes, sleep disorders and depression, with odds ratios ranging from 1.14 (sleep disorders) to 1.33 (depression) after adjustment for sex, age, social position and current unemployment (S12 Table). The overexposure to risk factors was associated with an increased prevalence of non-fatal myocardial infarction with an odds ratio of 1.31 after adjustment for sex, age, parental history of cardiovascular event, social position, current unemployment and risk factors (S13 Table). Participants with bad work environment who were unemployed at inclusion compared to those who were employed at that time were overexposed to at least four risk factors, including non-moderate alcohol consumption, smoking, leisure-time physical inactivity and depression, with odds ratios ranging from 1.19 (leisure-time physical inactivity) to 1.43 (smoking and depression) after adjustment for sex, age, social position and past unemployment (S14 Table). Like for social position, no significant interaction was found between work environment and past or current unemployment on the prevalence of risk factors and cardiovascular events (results not shown).

### Chronology of unemployment and cardiovascular events during the lifetime of participants

**Fig 1:**
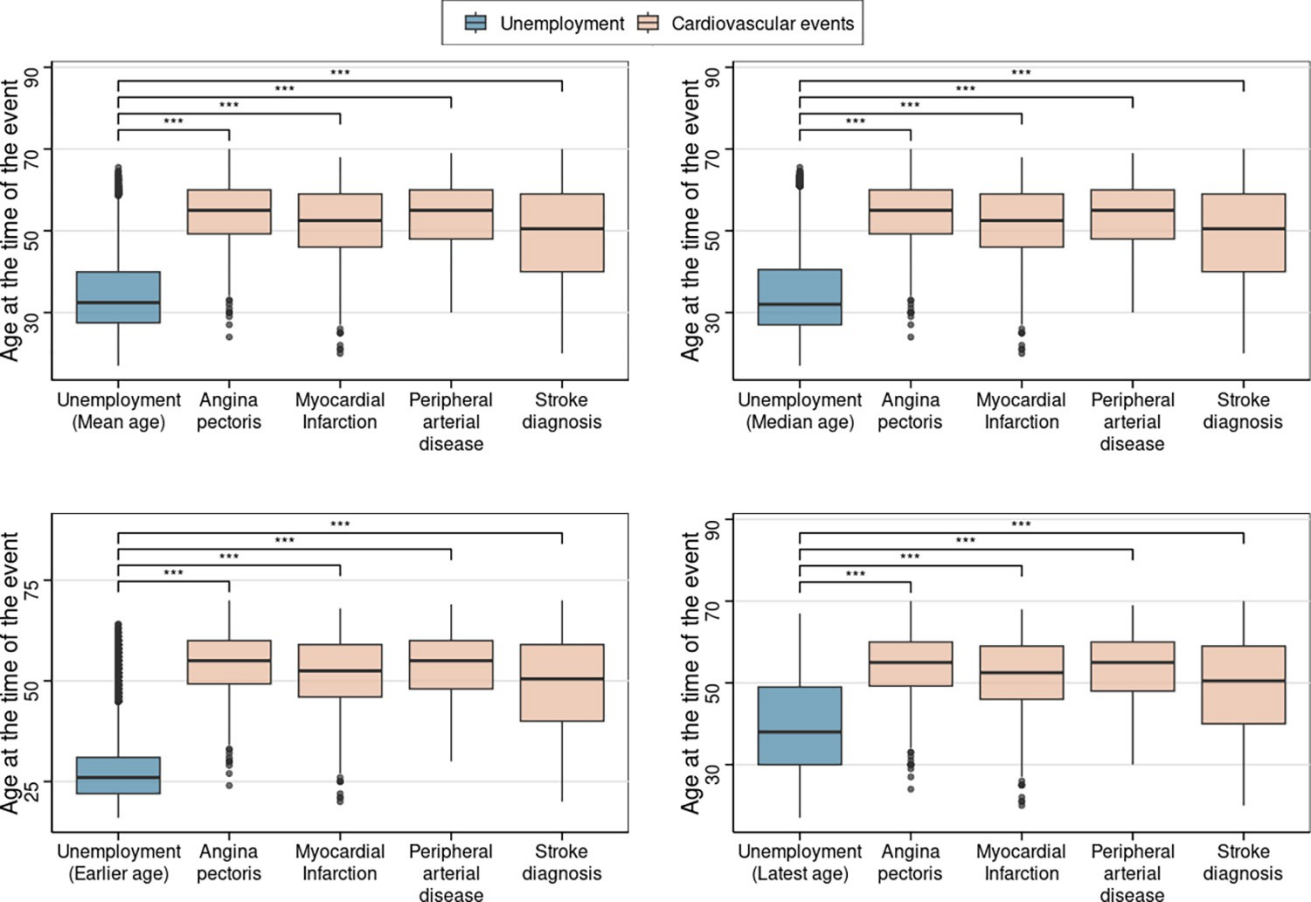
Age differences between the occurrence of unemployment episodes and cardiovascular events during the lifetime of participants. The age at which unemployment episodes occurred was expressed in four different ways: mean, median, earlier or latest age. In each box plot, the horizontal line represents the median value, the ends of the box represent the 1st and 3rd quartiles and the length of the box is the interquartile range, the lines on each end of the box extend to the outermost values that fall within 1st quartile -1.5*(interquartile range) and 3rd quartile + 1.5*(interquartile range), the values below or above these boundaries are shown as individual outliers. The differences were assessed with the non-parametric Wilcoxon-Mann-Whitney test. * p<0.05; ** p<0.01; *** p<0.001.

In order to test the possibility of reverse causation where cardiovascular events would have preceded unemployment, the age of participants at which unemployed quarters were declared was compared with the age at which cardiovascular events occurred. It appears that unemployment episodes popped up much earlier than cardiovascular events with a mean or median difference of approximately 15 to 20 years depending on the type of events. Thus, the mean and median ages at which the episodes occurred were respectively 34.4 and 34.7 years while they were 51.7 and 52.5 years for myocardial infarction and 53.8 and 55 years for peripheral arterial disease (Fig 1). Even the latest episodes occurred on average at 39.8 years, i.e., approximately 12 years before myocardial infarction and peripheral arterial disease.

## Discussion

The present study reports cardiovascular burden of people who are or have been unemployed, taking into account their social position and work environment. It was performed with data collected in a large population-based French cohort during the last decade. Unemployment appears to be associated with an overexposure to several cardiovascular risk factors including non-moderate alcohol consumption, smoking, leisure-time physical inactivity, obesity, diabetes, sleep disorders and depression, in agreement with results previously reported [8, 46–51]. Unemployment is also associated with increased rates of cardiovascular events, at least myocardial infarction and peripheral arterial disease in the present study, as suggested by prior reports [1–6]. However, the cardiovascular burden associated to current or past experience of unemployment is not the same. People who are currently unemployed are exposed to a smaller number of risk factors, mostly behavioral factors, compared to those who have been unemployed. In addition, this overexposure does not translate into an increased risk of cardiovascular events, which is expected as these events typically result from long-term exposure to risk factors. Importantly, the strength of the association between past unemployment and the prevalence of cardiovascular risk factors and events depends on the duration of unemployment, longer the duration during lifetime, higher the prevalence, as already suggested for the risk of myocardial infarction [2]. It is interesting to note that long-term unemployment remains associated with the prevalence of myocardial infarction and peripheral arterial disease even after adjustment for behavioral and clinical risk factors, suggesting that unemployment may increase cardiovascular risk not only by overexposure to common risk factors but also through other pathways yet to be defined. Identifying these pathways may not be so easy as the potential stressful effects of unemployment are numerous and entangled. For example, neuro-hormonal changes such as elevated cortisol levels might be involved to some extent among many others [3].

The need to take into account social position and work environment in order to assess the specific contribution of unemployment clearly appears from the present analyses. First, although work environment is not completely determined by social position, including occupation as working conditions can vary substantially for the same job [52], both are highly interrelated; the better one’s social position, the better one’s work environment tends to be [29]. Second, current and past unemployment are tightly associated with each other and are strongly linked to both social position and work environment. In these conditions, finding that unemployment is associated with the prevalence of cardiovascular risk factors and events after adjusting for social position and work environment strongly suggest an independent effect, especially when using global measures that thoroughly assess work environment and social position as in the present analyses [35]. An independent effect of unemployment is also supported by the fact that the association with the prevalence of risk factors and events is observed in people with low social position after adjustment for work environment and in those with bad work environment after adjustment for social position. It seems therefore that people with low social position, who as a consequence have high cardiovascular risk, are not less affected by unemployment than socially privileged individuals whose cardiovascular risk is low. The same conclusion can be made for people with bad work environment suggesting that even the worse working conditions are still better than unemployment when considering cardiovascular health, job exit not appearing like a salvation. This means that the independent effect of unemployment could add up to those of low social position and bad work environment during lifetime to further increase cardiovascular risk.

This study has several limitations. A first one is the external validity of the findings that were obtained in a cohort of participants who were not representative of the French population. A second one is that occupational and social data as well as health status were self-reported and may therefore have been relatively imprecise. A third one is the direct consequence of self-reporting that precluded the recording of fatal cardiovascular events. A fourth one is that social position and work environment were assessed at the time of the inclusion and may have not reflected the conditions in which participants lived during their lifetime, although a complete disconnection is unlikely. A fifth one is that total duration of past unemployment was available but not the number of unemployment episodes. Finally, due to the lack of prospective design of the analyses, reverse causation where poor health status of participants from the very beginning would have increased the risk of unemployment during their lifetime cannot be completely ruled out [53]. However, it is very unlikely given that unemployment occurred well prior to cardiovascular events.

In conclusion, this study suggests that unemployment can increase cardiovascular risk independently from social position and work environment. On the short term, it would do so mostly by increasing risky behaviors and causing depression. On the long term, these effects would translate into a high prevalence of clinical risk factors and consequently an increased incidence of cardiovascular events. Long-term unemployment seems to also increase cardiovascular risk independently of common risk factors through pathways that are yet to be defined. Long-term effects, which are stronger when unemployment lasts longer, are observed whatever social position and work environment and therefore could add up to those of low social position and bad work environment during lifetime to further increase cardiovascular risk. Unemployed people might benefit from a specific medical monitoring in general practice, especially if they are socially disadvantaged and/or had poor working conditions.

## Supporting information

S1 Fig Multiple correspondence analysis showing the association between the different indicators used to characterize social position of participants at inclusion. The plot uses the two first dimensions which explain respectively 18.5 and 11.5% of the total inertia (60.2 and 8.0% with Greenacre adjustment).
(DOCX)

S2 Fig Multiple correspondence analysis showing the association between the different occupational exposures used to characterize work environment of participants at inclusion. The plot uses the two first dimensions which explain respectively 16.8 and 12.0% of the total inertia (43.5 and 17.8% with Greenacre adjustment).
(DOCX)

S3 Fig Multiple correspondence analysis showing the association between social position, work environment and past or current unemployment at inclusion. The plot uses the two first dimensions which explain respectively 28.8 and 21.2% of the total inertia (47.9 and 35.3% with Greenacre adjustment).
(DOCX)

S1 Table Percentages of excluded participants for each cardiovascular risk factor. (DOCX)

S2 Table Characteristics of cohort participants with or without missing values compared to randomly selected individuals from the French population. (DOCX)

S3 Table Indicators of social position in participants at inclusion according to their past experience of unemployment. (DOCX)

S4 Table Indicators of work environment in participants at inclusion according to their past experience of unemployment. (DOCX)

S5 Table Adjusted odds ratios (95% confidence interval, p) for the prevalence of cardiovascular events in participants at inclusion according to their exposure to common risk factors. (DOCX)

S6 Table Cardiovascular risk factors in participants according to their current experience of unemployment. (DOCX)

S7 Table Adjusted odds ratios (95% confidence interval) for the prevalence of cardiovascular risk factors in participants at inclusion according to their current experience of unemployment. (DOCX)

S8 Table Adjusted odds ratios (95% confidence interval) for the prevalence of non-fatal cardiovascular events in participants at inclusion according to their current experience of unemployment. (DOCX)

S9 Table Adjusted odds ratios (95% confidence interval) for the prevalence of cardiovascular risk factors at inclusion in participants with low social position according to their past experience of unemployment. (DOCX)

S10 Table Adjusted odds ratios (95% confidence interval) for the prevalence of non-fatal myocardial infarction and peripheral arterial disease at inclusion in participants with low social position according to their past experience of unemployment. (DOCX)

S11 Table Adjusted odds ratios (95% confidence interval) for the prevalence of cardiovascular risk factors in participants at inclusion with low social position according to their current experience of unemployment. (DOCX)

S12 Table Adjusted odds ratios (95% confidence interval) for the prevalence of cardiovascular risk factors at inclusion in participants with bad work environment according to their past experience of unemployment. (DOCX)

S13 Table Adjusted odds ratios (95% confidence interval) for the prevalence of non-fatal myocardial infarction and peripheral arterial disease at inclusion in participants with bad work environment according to their past experience of unemployment. (DOCX)

S14 Table Adjusted odds ratios (95% confidence interval) for the prevalence of cardiovascular risk factors at inclusion in participants with bad work environment according to their current experience of unemployment. (DOCX)
