# Supplementary material for: Cardiovascular burden and unemployment: A retrospective study in a large population-based French cohort
Source: PLoS One. 2023 Jul 17;18(7):e0288747. doi: 10.1371/journal.pone.0288747 (PMC10351739; doi:10.1371/journal.pone.0288747)
Supplement: S1 Fig — The plot uses the two first dimensions which explain respectively 18.5 and 11.5% of the total inertia (60.2 and 8.0% with Greenacre adjustment). (DOCX) [file pone.0288747.s001.docx]

**S1 Fig:** Multiple correspondence analysis showing the association between the different indicators used to characterize social position of participants at inclusion. The plot uses the two first dimensions which explain respectively 18.5 and 11.5% of the total inertia (60.2 and 8.0% with Greenacre adjustment).
