# Supplementary material for: Cardiovascular burden and unemployment: A retrospective study in a large population-based French cohort
Source: PLoS One. 2023 Jul 17;18(7):e0288747. doi: 10.1371/journal.pone.0288747 (PMC10351739; doi:10.1371/journal.pone.0288747)
Supplement: S2 Fig — The plot uses the two first dimensions which explain respectively 16.8 and 12.0% of the total inertia (43.5 and 17.8% with Greenacre adjustment). (DOCX) [file pone.0288747.s002.docx]

**S2 Fig:** Multiple correspondence analysis showing the association between the different occupational exposures used to characterize work environment of participants at inclusion. The plot uses the two first dimensions which explain respectively 16.8 and 12.0% of the total inertia (43.5 and 17.8% with Greenacre adjustment).
