# Supplementary material for: Cardiovascular burden and unemployment: A retrospective study in a large population-based French cohort
Source: PLoS One. 2023 Jul 17;18(7):e0288747. doi: 10.1371/journal.pone.0288747 (PMC10351739; doi:10.1371/journal.pone.0288747)

**S3 Fig:** Multiple correspondence analysis showing the association between social position, work environment and past or current unemployment at inclusion. The plot uses the two first dimensions which explain respectively 28.8 and 21.2% of the total inertia (47.9 and 35.3% with Greenacre adjustment).


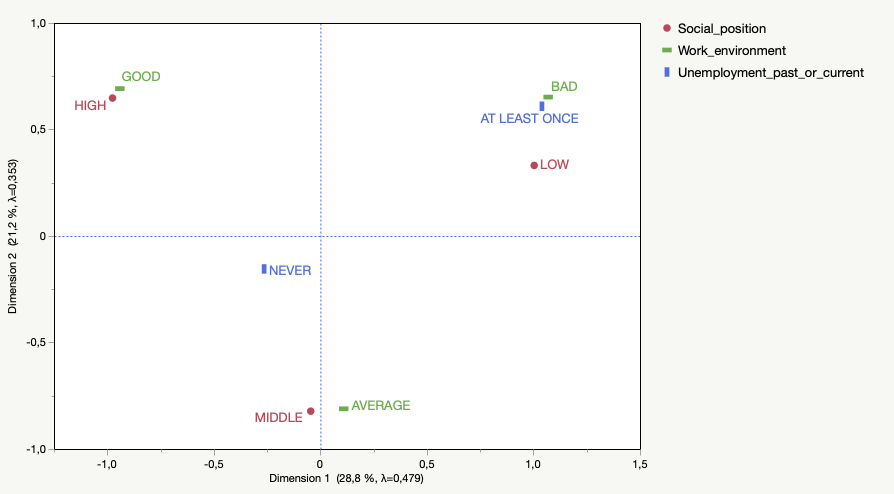

Supplement: S3 Fig — The plot uses the two first dimensions which explain respectively 28.8 and 21.2% of the total inertia (47.9 and 35.3% with Greenacre adjustment). (DOCX) [file pone.0288747.s003.docx]
