# Supplementary material for: Cardiovascular burden and unemployment: A retrospective study in a large population-based French cohort
Source: PLoS One. 2023 Jul 17;18(7):e0288747. doi: 10.1371/journal.pone.0288747 (PMC10351739; doi:10.1371/journal.pone.0288747)
Supplement: S1 Table — (DOCX) [file pone.0288747.s004.docx]

**S1 Table:** Percentages of excluded participants for each cardiovascular risk factor.

|  | | **Whole**  **cohort** | **Excluded**  **participants** | | **SMD** |
| --- | --- | --- | --- | --- | --- |
|  |  | **n** | **n** | **%** |  |
| - | **All** | 205,203 | 74,017 | 36.1 | - |
| **Sex** | **Women** | 110,193 | 42,950 | 39.0 | 0.131 |
|  | **Men** | 95,010 | 31,067 | 32.7 |  |
| **Age (y)** | **18-39** | 64,452 | 18,961 | 29.4 | 0.198 |
|  | **40-54** | 69,341 | 23,927 | 34.5 |  |
|  | **55-75** | 71,410 | 31,129 | 43.6 |  |
| **Parental history of**  **cardiovascular event** | **No** | 117,620 | 42,202 | 35.9 | 0.001 |
|  | **Yes** | 86,990 | 31,222 | 35.9 |  |
| **Past**  **unemployment** | **Never** | 171,177 | 59,006 | 34.5 | 0.198 |
|  | **At least once** | 34,026 | 15,011 | 44.1 |  |
| **Current**  **unemployment** | **No** | 190,719 | 67,811 | 35.6 | 0.150 |
|  | **Yes** | 14,484 | 6206 | 42.8 |  |
| **Social position** | **High** | 52,480 | 14,269 | 27.2 | 0.302 |
|  | **Middle** | 93,602 | 30,907 | 33.0 |  |
|  | **Low** | 59,089 | 28,809 | 48.8 |  |
| **Work environment** | **Good** | 64,639 | 19,300 | 29.9 | 0.197 |
|  | **Average** | 93,208 | 33,898 | 36.4 |  |
|  | **Bad** | 47,356 | 20,819 | 44.0 |  |
| **Lifetime non-moderate**  **alcohol consumption** | **Rarely** | 34,396 | 15,875 | 46.2 | 0.255 |
|  | **Sometimes** | 38,616 | 11,229 | 29.1 |  |
|  | **Often** | 118,465 | 33,187 | 28.0 |  |
| **Smoking** | **Never** | 94,017 | 33,465 | 35.6 | 0.071 |
|  | **Former** | 66,448 | 21,001 | 31.6 |  |
|  | **Current** | 36,297 | 11,110 | 30.6 |  |
| **Leisure-time**  **physical inactivity** | **No** | 178,957 | 59,812 | 33.4 | 0.078 |
|  | **Yes** | 19,154 | 7113 | 37.1 |  |
| **Body mass index** | **Optimal** | 114,486 | 37,302 | 32.6 | 0.125 |
|  | **Overweight** | 61,373 | 21,996 | 35.8 |  |
|  | **Obese** | 25,048 | 10,423 | 41.6 |  |
| **Hypertension** | **No** | 176,603 | 58,309 | 33.0 | 0.218 |
|  | **Yes** | 22,846 | 9954 | 43.6 |  |
| **Dyslipidemia** | **No** | 182,005 | 60,105 | 33.0 | 0.202 |
|  | **Yes** | 16,230 | 6944 | 42.8 |  |
| **Diabetes** | **No** | 194,514 | 65,278 | 33.6 | 0.353 |
|  | **Yes** | 3958 | 2008 | 50.7 |  |
| **Sleep disorders** | **No** | 73,595 | 25,468 | 34.6 | 0.018 |
|  | **Yes** | 128,679 | 45,620 | 35.5 |  |
| **Depression** | **No** | 143,198 | 30,719 | 21.5 | 0.164 |
|  | **Yes** | 26,170 | 7463 | 28.5 |  |

# Differences in the percentages of excluded participants between levels of each risk factor were assessed by computing standardized mean differences (SMD).
