# Supplementary material for: Cardiovascular burden and unemployment: A retrospective study in a large population-based French cohort
Source: PLoS One. 2023 Jul 17;18(7):e0288747. doi: 10.1371/journal.pone.0288747 (PMC10351739; doi:10.1371/journal.pone.0288747)
Supplement: S3 Table — (DOCX) [file pone.0288747.s006.docx]

# **S3 Table:** Indicators of social position in participants at inclusion according to their past experience of unemployment.

|  | | **Past unemployment** | | | | **SMD** |
| --- | --- | --- | --- | --- | --- | --- |
|  |  | **Never** | | **At least once** | |  |
|  |  | **n** | **%** | **n** | **%** |  |
| **Education (y)** | **≥17** | 31,737 | 88.6 | 4071 | 11.4 | 0.250 |
|  | **14-16** | 41,530 | 87.0 | 6195 | 13.0 |  |
|  | **12-13** | 17,368 | 83.1 | 3541 | 16.9 |  |
|  | **≤11** | 20,588 | 80.3 | 5062 | 19.7 |  |
| **Occupation** | **Management** | 37,843 | 77.9 | 4573 | 10.8 | 0.410 |
|  | **Intermediate** | 32,078 | 88.7 | 4076 | 11.3 |  |
|  | **Blue collar/clerk** | 31,025 | 89.1 | 8807 | 22.1 |  |
| **Income** | **Very high** | 36,455 | 90.6 | 3801 | 9.4 | 0.379 |
|  | **High** | 35,052 | 86.1 | 5646 | 13.9 |  |
|  | **Middle** | 25,789 | 81.6 | 5803 | 18.4 |  |
|  | **Low** | 8276 | 74.6 | 2816 | 25.4 |  |
| **Spouse occupation** | **Management** | 28,172 | 87.3 | 4108 | 12.7 | 0.136 |
|  | **Intermediate** | 22,105 | 87.6 | 3119 | 12.4 |  |
|  | **Blue collar/clerk** | 29,905 | 84.1 | 5658 | 15.9 |  |
| **Social vulnerability** | **Low** | 36,125 | 79.2 | 4303 | 20.8 | 0.271 |
|  | **Average** | 39,865 | 85.6 | 6329 | 14.4 |  |
|  | **High** | 35,752 | 90.4 | 8306 | 9.6 |  |

# The percentages were calculated relatively to the number of participants in each indicator level; the differences between past unemployment experiences were assessed by computing standardized mean differences (SMD).
