# Supplementary material for: Cardiovascular burden and unemployment: A retrospective study in a large population-based French cohort
Source: PLoS One. 2023 Jul 17;18(7):e0288747. doi: 10.1371/journal.pone.0288747 (PMC10351739; doi:10.1371/journal.pone.0288747)
Supplement: S4 Table — (DOCX) [file pone.0288747.s007.docx]

# **S4 Table:** Indicators of work environment in participants at inclusion according to their past experience of unemployment.

|  | | **Past unemployment** | | | | **SMD** |
| --- | --- | --- | --- | --- | --- | --- |
|  |  | **Never** | | **At least once** | |  |
|  |  | **n** | **%** | **n** | **%** |  |
| **Commuting time** | **<1h** | 63,137 | 86.4 | 9961 | 13.6 | 0.036 |
|  | **1h-2h** | 16,309 | 86.3 | 2582 | 13.7 |  |
|  | **>2h** | 3087 | 84.1 | 585 | 15.9 |  |
| **Clocking in and out** | **No** | 64,926 | 86.8 | 9847 | 13.2 | 0.090 |
|  | **Yes** | 17,980 | 84.2 | 3368 | 15.8 |  |
| **Regular working hours** | **No** | 46,333 | 86.7 | 7098 | 13.3 | 0.044 |
|  | **Yes** | 36,895 | 85.7 | 6173 | 14.3 |  |
| **Long working hours** | **No** | 78,934 | 85.6 | 13,317 | 14.4 | 0.007 |
|  | **Yes** | 33,237 | 85.4 | 5698 | 14.6 |  |
| **Night work** | **No** | 101,805 | 85.6 | 17,106 | 14.4 | 0.027 |
|  | **Yes** | 10,366 | 84.4 | 1909 | 15.6 |  |
| **Dealing with the public** | **No** | 40,817 | 85.9 | 6682 | 14.1 | 0.030 |
|  | **Yes** | 16,421 | 86.7 | 2514 | 13.3 |  |
| **Driving on public road** | **No** | 73,600 | 86.3 | 11,646 | 13.7 | 0.041 |
|  | **Yes** | 9729 | 84.8 | 1742 | 15.2 |  |
| **Repetitive work** | **No** | 63,239 | 87.4 | 9127 | 12.6 | 0.170 |
|  | **Yes** | 18,594 | 82.5 | 3950 | 17.5 |  |
| **Working with a screen** | **No** | 18,774 | 83.4 | 3729 | 16.6 | 0.124 |
|  | **Yes** | 64,198 | 87.0 | 9580 | 13.0 |  |
| **Standing work posture** | **No** | 44,006 | 85.8 | 7270 | 14.2 | 0.029 |
|  | **Yes** | 39,403 | 86.5 | 6142 | 13.5 |  |
| **Handling heavy loads** | **No** | 52,308 | 87.1 | 7751 | 12.9 | 0.102 |
|  | **Yes** | 30,765 | 84.6 | 5612 | 15.4 |  |
| **Physically demanding work** | **No** | 82,601 | 87.3 | 12,049 | 12.7 | 0.223 |
|  | **Yes** | 29,570 | 83.3 | 6966 | 19.1 |  |
| **Exposure to vibrations** | **No** | 79,902 | 86.4 | 12,724 | 13.7 | 0.043 |
|  | **Yes** | 2761 | 83.4 | 552 | 16.7 |  |
| **Exposure to noise** | **No** | 80,054 | 86.3 | 12,638 | 16.6 | 0.106 |
|  | **Yes** | 32,117 | 82.0 | 6377 | 16.6 |  |
| **Outdoor work** | **No** | 76,426 | 86.4 | 11,995 | 13.6 | 0.073 |
|  | **Yes** | 7966 | 83.4 | 1585 | 16.6 |  |
| **Working in the cold** | **No** | 80,968 | 86.3 | 12,833 | 13.7 | 0.060 |
|  | **Yes** | 2535 | 82.0 | 556 | 18.0 |  |
| **Working in the heat** | **No** | 80,098 | 86.3 | 12,682 | 13.7 | 0.054 |
|  | **Yes** | 3554 | 83.0 | 726 | 17.0 |  |
| **Exposure to chemicals** | **No** | 75,626 | 86.8 | 11,499 | 13.2 | 0.145 |
|  | **Yes** | 36,545 | 82.9 | 7516 | 17.1 |  |
| **Effort-reward imbalance** | **Low** | 26,366 | 86.2 | 4234 | 13.8 | 0.083 |
|  | **Average** | 31,924 | 87.6 | 4514 | 12.4 |  |
|  | **High** | 24,853 | 85.3 | 4285 | 14.7 |  |

# The percentages were calculated relatively to the number of participants in each indicator level; the differences between past unemployment experiences were assessed by computing standardized mean differences (SMD).
