# Supplementary material for: Cardiovascular burden and unemployment: A retrospective study in a large population-based French cohort
Source: PLoS One. 2023 Jul 17;18(7):e0288747. doi: 10.1371/journal.pone.0288747 (PMC10351739; doi:10.1371/journal.pone.0288747)
Supplement: S5 Table — (DOCX) [file pone.0288747.s008.docx]

**S5 Table:** Adjusted odds ratios (95% confidence interval, p) for the prevalence of cardiovascular events in participants at inclusion according to their exposure to common risk factors.

|  | | **Stroke** | | **Myocardial infarction** | | **Angina pectoris** | | **Peripheral arterial disease** | |
| --- | --- | --- | --- | --- | --- | --- | --- | --- | --- |
| **Sex** | **Women** | 1.00 |  | 1.00 |  | 1.00 |  | 1.00 |  |
|  | **Men** | 1.09 (0.94-1.25) | 0.26 | 4.72 (3.82-5.83) | <0.0001 | 3.46 (2.82-4.25) | <0.0001 | 2.47 (1.80-3.40) | <0.0001 |
| **Age (y)** | **18-39** | 1.00 |  | 1.00 |  | 1.00 |  | 1.00 |  |
|  | **40-54** | 2.58 (1.96-3.39) | <0.0001 | 2.74 (1.77-4.25) | <0.0001 | 3.66 (2.15-6.24) | <0.0001 | 1.80 (0.98-3.31) | 0.06 |
|  | **55-75** | 4.41 (3.37-5.78) | <0.0001 | 6.35 (4.17-9.67) | <0.0001 | 8.87 (5.30-14.9) | <0.0001 | 4.76 (2.69-8.44) | <0.0001 |
| **Parental history of**  **cardiovascular event** | **No** | 1.00 |  | 1.00 |  | 1.00 |  | 1.00 |  |
|  | **Yes** | 0.93 (0.81-1.08) | 0.36 | 1.62 (1.40-1.87) | <0.0001 | 1.75 (1.50-2.03) | <0.0001 | 1.07 (0.83-1.38) | 0.60 |
| **Lifetime non-moderate**  **alcohol consumption** | **Rarely** | 1.00 |  | 1.00 |  | 1.00 |  | 1.00 |  |
|  | **Sometimes** | 1.01 (0.80-1.27) | 0.94 | 0.78 (0.60-1.03) | 0.09 | 1.12 (0.82-1.53) | 0.49 | 0.74 (0.46-1.20) | 0.22 |
|  | **Often** | 0.95 (0.77-1.16) | 0.59 | 0.70 (0.56-0.89) | 0.003 | 1.07 (0.82-1.41) | 0.60 | 0.70 (0.48-1.04) | 0.08 |
| **Smoking** | **Never** | 1.00 |  | 1.00 |  | 1.00 |  | 1.00 |  |
|  | **Former** | 1.02 (0.88-1.19) | 0.75 | 1.80 (1.52-2.14) | <0.0001 | 1.41 (1.19-1.67) | <0.0001 | 3.65 (2.47-5.40) | <0.0001 |
|  | **Current** | 1.05 (0.86-1.29) | 0.62 | 1.52 (1.20-1.93) | 0.0005 | 1.02 (0.79-1.33) | 0.86 | 7.53 (4.96-11.4) | <0.0001 |
| **Leisure-time**  **physical inactivity** | **No** | 1.00 |  | 1.00 |  | 1.00 |  | 1.00 |  |
|  | **Yes** | 1.18 (0.95-1.48) | 0.14 | 1.05 (0.83-1.34) | 0.69 | 0.90 (0.68-1.19) | 0.46 | 0.99 (0.64-1.51) | 0.95 |
| **Body mass index** | **Optimal** | 1.00 |  | 1.00 |  | 1.00 |  | 1.00 |  |
|  | **Overweight** | 1.08 (0.93-1.26) | 0.30 | 1.12 (0.94-1.33) | 0.21 | 0.98 (0.82-1.18) | 0.85 | 0.78 (0.58-1.05) | 0.10 |
|  | **Obesity** | 0.92 (0.75-1.12) | 0.39 | 1.18 (0.96-1.45) | 0.12 | 0.86 (0.69-1.08) | 0.19 | 0.60 (0.42-0.87) | 0.008 |
| **Hypertension** | **No** | 1.00 |  | 1.00 |  | 1.00 |  | 1.00 |  |
|  | **Yes** | 3.15 (2.69-3.68) | <0.0001 | 1.98 (1.69-2.32) | <0.0001 | 2.63 (2.22-3.11) | <0.0001 | 4.44 (3.32-5.94) | <0.0001 |
| **Dyslipidemia** | **No** | 1.00 |  | 1.00 |  | 1.00 |  | 1.00 |  |
|  | **Yes** | 3.19 (2.72-3.74) | <0.0001 | 7.62 (6.47-8.98) | <0.0001 | 7.03 (5.92-8.35) | <0.0001 | 4.38 (3.29-5.84) | <0.0001 |
| **Diabetes** | **No** | 1.00 |  | 1.00 |  | 1.00 |  | 1.00 |  |
|  | **Yes** | 0.75 (0.55-1.03) | 0.08 | 1.28 (1.02-1.61) | 0.03 | 1.10 (0.85-1.42) | 0.47 | 1.56 (1.06-2.29) | 0.02 |
| **Sleep disorders** | **No** | 1.00 |  | 1.00 |  | 1.00 |  | 1.00 |  |
|  | **Yes** | 1.09 (0.94-1.25) | 0.26 | 1.27 (1.09-1.48) | 0.002 | 1.22 (1.04-1.44) | 0.02 | 1.06 (0.81-1.37) | 0.68 |
| **Depression** | **No** | 1.00 |  | 1.00 |  | 1.00 |  | 1.00 |  |
|  | **Yes** | 1.39 (1.17-1.66) | 0.0002 | 1.23 (1.00-1.51) | 0.05 | 1.44 (1.16-1.78) | 0.0008 | 1.20 (0.85-1.70) | 0.30 |

For each cardiovascular event, odds ratios were computed with models including sex, age, parental history of cardiovascular event, lifetime non-moderate alcohol consumption, smoking, leisure-time physical inactivity, body mass index, hypertension, dyslipidemia, diabetes, sleep disorders and depression.
