# Supplementary material for: Cardiovascular burden and unemployment: A retrospective study in a large population-based French cohort
Source: PLoS One. 2023 Jul 17;18(7):e0288747. doi: 10.1371/journal.pone.0288747 (PMC10351739; doi:10.1371/journal.pone.0288747)
Supplement: S6 Table — (DOCX) [file pone.0288747.s009.docx]

# **S6 Table:** Cardiovascular risk factors in participants according to their current experience of unemployment.

|  | | **Current unemployment** | | | | **SMD** |
| --- | --- | --- | --- | --- | --- | --- |
|  |  | **No**  **(n=122,908)** | | **Yes**  **(n=8278)** | |  |
|  |  | **n** | **%** | **n** | **%** |  |
| **Sex** | **Women** | 62,737 | 93.3 | 4506 | 6.7 | 0.068 |
|  | **Men** | 60,171 | 94.1 | 3772 | 5.9 |  |
| **Age (y)** | **18-39** | 38,429 | 90.7 | 3940 | 9.3 | 0.732 |
|  | **40-54** | 41,994 | 94.2 | 2575 | 5.8 |  |
|  | **55-75** | 42,485 | 96.0 | 1763 | 4.0 |  |
| **Parental history of**  **cardiovascular event** | **No** | 93,020 | 58.6 | 6563 | 41.4 | 0.086 |
|  | **Yes** | 29,888 | 94.6 | 1715 | 5.4 |  |
| **Past unemployment** | **No** | 107,631 | 96.0 | 4540 | 4.0 | 0.775 |
|  | **Yes** | 15,277 | 80.3 | 3738 | 19.7 |  |
| **Social position** | **High** | 37,238 | 97.5 | 973 | 2.5 | 0.579 |
|  | **Middle** | 58,975 | 94.1 | 3720 | 5.9 |  |
|  | **Low** | 26,695 | 88.2 | 3585 | 11.8 |  |
| **Work environment** | **Good** | 40,261 | 97.6 | 978 | 2.4 | 0.904 |
|  | **Average** | 45,341 | 97.0 | 1388 | 3.0 |  |
|  | **Bad** | 37,306 | 86.3 | 5912 | 13.7 |  |
| **Lifetime non-moderate**  **alcohol consumption** | **Rarely** | 17,013 | 91.9 | 1508 | 8.1 | 0.121 |
|  | **Sometimes** | 25,694 | 93.8 | 1693 | 6.2 |  |
|  | **Often** | 80,201 | 94.0 | 5077 | 6.0 |  |
| **Smoking** | **Never** | 57,456 | 94.9 | 3096 | 5.1 | 0.333 |
|  | **Former** | 42,960 | 63.3 | 2487 | 36.7 |  |
|  | **Current** | 22,492 | 89.3 | 2695 | 10.7 |  |
| **Leisure-time**  **physical inactivity** | **No** | 111,785 | 93.8 | 7360 | 6.2 | 0.068 |
|  | **Yes** | 11,123 | 92.4 | 918 | 7.6 |  |
| **Body mass index** | **Optimal** | 72,241 | 93.6 | 4943 | 6.4 | 0.098 |
|  | **Overweight** | 37,161 | 94.4 | 2216 | 5.6 |  |
|  | **Obesity** | 13,506 | 92.3 | 1119 | 7.7 |  |
| **Hypertension** | **No** | 110,574 | 93.6 | 7498 | 6.4 | 0.006 |
|  | **Yes** | 12,334 | 94.1 | 780 | 5.9 |  |
| **Dyslipidemia** | **No** | 114,004 | 93.7 | 7698 | 6.3 | 0.031 |
|  | **Yes** | 8904 | 93.9 | 580 | 6.1 |  |
| **Diabetes** | **No** | 121,072 | 93.7 | 8164 | 6.3 | 0.010 |
|  | **Yes** | 1836 | 94.2 | 114 | 5.8 |  |
| **Sleep** **disorders** | **No** | 45,528 | 94.6 | 2599 | 5.4 | 0.119 |
|  | **Yes** | 77,380 | 57.7 | 5679 | 42.3 |  |
| **Depression** | **No** | 106,380 | 63.6 | 6099 | 36.4 | 0.327 |
|  | **Yes** | 16,528 | 88.4 | 2179 | 11.6 |  |

# The percentages were calculated relatively to the number of participants in each risk factor level; the differences between current unemployment experiences were assessed by computing standardized mean differences (SMD).
