# Supplementary material for: Cardiovascular burden and unemployment: A retrospective study in a large population-based French cohort
Source: PLoS One. 2023 Jul 17;18(7):e0288747. doi: 10.1371/journal.pone.0288747 (PMC10351739; doi:10.1371/journal.pone.0288747)
Supplement: S7 Table — (DOCX) [file pone.0288747.s010.docx]

**S7 Table:** Adjusted odds ratios (95% confidence interval) for the prevalence of cardiovascular risk factors in participants at inclusion according to their current experience of unemployment.

|  | **Current unemployment** | **n** | **%** | **Models 1** | **p** | **Models 2** | **p** |
| --- | --- | --- | --- | --- | --- | --- | --- |
| **Non-moderate**  **alcohol consumption** | **No** | 12,582 | 10.2 | 1.00 |  | 1.00 |  |
|  | **Yes** | 1241 | 15.0 | 1.61 (1.51-1.71) | <0.0001 | 1.37 (1.28-1.46) | <0.0001 |
| **Smoking** | **No** | 22,492 | 18.3 | 1.00 |  | 1.00 |  |
|  | **Yes** | 2695 | 32.6 | 1.93 (1.83-2.02) | <0.0001 | 1.55 (1.47-1.64) | <0.0001 |
| **Leisure-time**  **physical inactivity** | **No** | 11,123 | 9.0 | 1.00 |  | 1.00 |  |
|  | **Yes** | 918 | 11.1 | 1.18 (1.10-1.27) | <0.0001 | 1.19 (1.11-1.29) | <0.0001 |
| **Obesity** | **No** | 13,506 | 11.0 | 1.00 |  | 1.00 |  |
|  | **Yes** | 1119 | 13.5 | 1.46 (1.36-1.56) | <0.0001 | 1.09 (1.01-1.17) | 0.02 |
| **Hypertension** | **No** | 12,334 | 10.0 | 1.00 |  | 1.00 |  |
|  | **Yes** | 558 | 6.7 | 0.94 (0.86-1.03) | 0.21 | 0.99 (0.92-1.06) | 0.70 |
| **Dyslipidemia** | **No** | 8904 | 7.2 | 1.00 |  | 1.00 |  |
|  | **Yes** | 382 | 4.6 | 0.93 (0.83-1.04) | 0.18 | 0.96 (0.89-1.05) | 0.37 |
| **Diabetes** | **No** | 1836 | 1.5 | 1.00 |  | 1.00 |  |
|  | **Yes** | 114 | 1.4 | 1.39 (1.14-1.69) | 0.0009 | 1.00 (0.82-1.23) | 0.99 |
| **Sleep disorders** | **No** | 77,380 | 63.0 | 1.00 |  | 1.00 |  |
|  | **Yes** | 5679 | 68.6 | 1.25 (1.19-1.31) | <0.0001 | 1.04 (0.99-1.10) | 0.10 |
| **Depression** | **No** | 16,528 | 13.4 | 1.00 |  | 1.00 |  |
|  | **Yes** | 2179 | 26.3 | 2.25 (2.13-2.37) | <0.0001 | 1.58 (1.49-1.67) | <0.0001 |

The percentages were calculated relatively to the number of participants in each current experience of unemployment (no=122,908; yes=8278).

Models 1 were adjusted for sex and age.

Models 2 were adjusted for sex, age, past unemployment, social position and work environment.
