# Supplementary material for: Cardiovascular burden and unemployment: A retrospective study in a large population-based French cohort
Source: PLoS One. 2023 Jul 17;18(7):e0288747. doi: 10.1371/journal.pone.0288747 (PMC10351739; doi:10.1371/journal.pone.0288747)
Supplement: S8 Table — (DOCX) [file pone.0288747.s011.docx]

**S8 Table:** Adjusted odds ratios (95% confidence interval) for the prevalence of non-fatal cardiovascular events in participants at inclusion according to their current experience of unemployment.

|  | **Current unemployment** | **n** | **%** | **Models 1** | **p** | **Models 2** | **p** | **Models 3** | **p** |
| --- | --- | --- | --- | --- | --- | --- | --- | --- | --- |
| **Stroke** | **No** | 858 | 0.70 | 1.00 |  | 1.00 |  | 1.00 |  |
|  | **Yes** | 52 | 0.63 | 1.21 (0.91-1.60) | 0.19 | 1.01 (0.75-1.36) | 0.92 | 1.13 (0.84-1.52) | 0.42 |
| **Myocardial**  **infraction** | **No** | 791 | 0.64 | 1.00 |  | 1.00 |  | 1.00 |  |
|  | **Yes** | 39 | 0.47 | 1.09 (0.79-1.51) | 0.59 | 0.77 (0.55-1.08) | 0.14 | 0.91 (0.64-1.29) | 0.60 |
| **Angina pectoris** | **No** | 703 | 0.57 | 1.00 |  | 1.00 |  | 1.00 |  |
|  | **Yes** | 32 | 0.39 | 1.02 (0.71-1.46) | 0.90 | 0.76 (0.52-1.10) | 0.14 | 0.92 (0.63-1.35) | 0.67 |
| **Peripheral**  **arterial disease** | **No** | 245 | 0.20 | 1.00 |  | 1.00 |  | 1.00 |  |
|  | **Yes** | 17 | 0.21 | 1.48 (0.90-2.43) | 0.12 | 0.92 (0.55-1.55) | 0.76 | 0.95 (0.56-1.61) | 0.86 |

The percentages were calculated relatively to the number of participants in each current experience of unemployment (no=122,908; yes=8278).

Models 1 were adjusted for sex, age and parental history of cardiovascular event.

Models 2 were adjusted for sex, age, parental history of cardiovascular event, past unemployment, social position and work environment.

Models 3 were adjusted for sex, age, parental history of cardiovascular event, past unemployment, social position, work environment, lifetime alcohol consumption, smoking, leisure-time physical inactivity, obesity, hypertension, dyslipidemia, diabetes, sleep disorders and depression.
