# Supplementary material for: Cardiovascular burden and unemployment: A retrospective study in a large population-based French cohort
Source: PLoS One. 2023 Jul 17;18(7):e0288747. doi: 10.1371/journal.pone.0288747 (PMC10351739; doi:10.1371/journal.pone.0288747)
Supplement: S9 Table — (DOCX) [file pone.0288747.s012.docx]

**S9 Table:** Adjusted odds ratios (95% confidence interval) for the prevalence of cardiovascular risk factors at inclusion in participants with low social position according to their past experience of unemployment.

|  | **Past unemployment** | **n** | **%** | **Models 1** | **p** | **Models 2** | **p** |
| --- | --- | --- | --- | --- | --- | --- | --- |
| **Non-moderate**  **alcohol consumption** | **Never** | 2970 | 12.4 | 1.00 |  | 1.00 |  |
|  | **At least once** | 803 | 12.7 | 1.10 (1.01-1.20) | 0.02 | 1.03 (0.94-1.12) | 0.52 |
| **Smoking** | **Never** | 5934 | 24.7 | 1.00 |  | 1.00 |  |
|  | **At least once** | 1846 | 29.3 | 1.32 (1.24-1.41) | <0.0001 | 1.20 (1.13-1.29) | <0.0001 |
| **Leisure-time**  **physical inactivity** | **Never** | 2564 | 10.7 | 1.00 |  | 1.00 |  |
|  | **At least once** | 727 | 11.5 | 1.07 (0.98-1.17) | 0.11 | 1.05 (0.96-1.15) | 0.28 |
| **Obesity** | **Never** | 3976 | 16.6 | 1.00 |  | 1.00 |  |
|  | **At least once** | 1234 | 19.6 | 1.18 (1.10-1.27) | <0.0001 | 1.13 (1.05-1.22) | 0.001 |
| **Diabetes** | **Never** | 699 | 2.9 | 1.00 |  | 1.00 |  |
|  | **At least once** | 194 | 3.1 | 1.17 (0.99-1.38) | 0.07 | 1.14 (0.96-1.35) | 0.14 |
| **Sleep disorders** | **Never** | 15,732 | 65.6 | 1.00 |  | 1.00 |  |
|  | **At least once** | 4373 | 69.4 | 1.17 (1.10-1.24) | <0.0001 | 1.14 (1.07-1.21) | <0.0001 |
| **Depression** | **Never** | 4731 | 19.7 | 1.00 |  | 1.00 |  |
|  | **At least once** | 1699 | 27.0 | 1.42 (1.33-1.52) | <0.0001 | 1.30 (1.21-1.39) | <0.0001 |

The percentages were calculated relatively to the number of participants with low social position in each past experience of unemployment (never=23,977; at least once=6303).

Models 1 were adjusted for sex and age.

Models 2 were adjusted for sex, age, current unemployment and work environment.
