# Supplementary material for: Cardiovascular burden and unemployment: A retrospective study in a large population-based French cohort
Source: PLoS One. 2023 Jul 17;18(7):e0288747. doi: 10.1371/journal.pone.0288747 (PMC10351739; doi:10.1371/journal.pone.0288747)
Supplement: S10 Table — (DOCX) [file pone.0288747.s013.docx]

**S10 Table:** Adjusted odds ratios (95% confidence interval) for the prevalence of non-fatal myocardial infarction and peripheral arterial disease at inclusion in participants with low social position according to their past experience of unemployment.

|  | **Past unemployment** | **n** | **%** | **Models 1** | **p** | **Models 2** | **p** | **Models 3** | **p** |
| --- | --- | --- | --- | --- | --- | --- | --- | --- | --- |
| **Myocardial**  **infraction** | **Never** | 247 | 1.03 | 1.00 |  | 1.00 |  | 1.00 |  |
|  | **At least once** | 84 | 1.33 | 1.55 (1.20-2.00) | 0.0008 | 1.56 (1.20-2.02) | 0.0009 | 1.50 (1.15-1.97) | 0.003 |
| **Peripheral**  **arterial disease** | **Never** | 102 | 0.43 | 1.00 |  | 1.00 |  | 1.00 |  |
|  | **At least once** | 28 | 0.44 | 1.20 (0.79-1.83) | 0.40 | 1.14 (0.74-1.77) | 0.55 | 1.06 (0.68-1.65) | 0.80 |

The percentages were calculated relatively to the number of participants with low social position in each past experience of unemployment (never=23,977; at least once=6303).

Models 1 were adjusted for sex, age and parental history of cardiovascular event.

Models 2 were adjusted for sex, age, parental history of cardiovascular event, current unemployment and work environment.

Models 3 were adjusted for sex, age, parental history of cardiovascular event, current unemployment, work environment, lifetime alcohol consumption, smoking, leisure-time physical inactivity, obesity, hypertension, dyslipidemia, diabetes, sleep disorders and depression.
