# Supplementary material for: Cardiovascular burden and unemployment: A retrospective study in a large population-based French cohort
Source: PLoS One. 2023 Jul 17;18(7):e0288747. doi: 10.1371/journal.pone.0288747 (PMC10351739; doi:10.1371/journal.pone.0288747)
Supplement: S11 Table — (DOCX) [file pone.0288747.s014.docx]

**S11 Table:** Adjusted odds ratios (95% confidence interval) for the prevalence of cardiovascular risk factors in participants at inclusion with low social position according to their current experience of unemployment.

|  | **Current unemployment** | **n** | **%** | **Models 1** | **p** | **Models 2** | **p** |
| --- | --- | --- | --- | --- | --- | --- | --- |
| **Non-moderate**  **alcohol consumption** | **No** | 3206 | 12.0 | 1.00 |  | 1.00 |  |
|  | **Yes** | 567 | 15.8 | 1.44 (1.31-1.60) | <0.0001 | 1.36 (1.22-1.52) | <0.0001 |
| **Smoking** | **No** | 6310 | 23.6 | 1.00 |  | 1.00 |  |
|  | **Yes** | 1470 | 41.0 | 1.65 (1.53-1.78) | <0.0001 | 1.52 (1.40-1.65) | <0.0001 |
| **Leisure-time**  **physical inactivity** | **No** | 2796 | 10.5 | 1.00 |  | 1.00 |  |
|  | **Yes** | 495 | 13.8 | 1.12 (1.01-1.25) | 0.03 | 1.18 (1.05-1.32) | 0.005 |
| **Obesity** | **No** | 4545 | 17.0 | 1.00 |  | 1.00 |  |
|  | **Yes** | 665 | 18.5 | 1.34 (1.22-1.47) | <0.0001 | 1.21 (1.10-1.34) | 0.0001 |
| **Depression** | **No** | 5236 | 19.6 | 1.00 |  | 1.00 |  |
|  | **Yes** | 1194 | 33.3 | 1.81 (1.67-1.96) | <0.0001 | 1.44 (1.32-1.57) | <0.0001 |

The percentages were calculated relatively to the number of participants with low social position in each current experience of unemployment (no=26,695; yes=3585).

Models 1 were adjusted for sex and age.

Models 2 were adjusted for sex, age, past unemployment and work environment.
