# Supplementary material for: Cardiovascular burden and unemployment: A retrospective study in a large population-based French cohort
Source: PLoS One. 2023 Jul 17;18(7):e0288747. doi: 10.1371/journal.pone.0288747 (PMC10351739; doi:10.1371/journal.pone.0288747)
Supplement: S12 Table — (DOCX) [file pone.0288747.s015.docx]

**S12 Table:** Adjusted odds ratios (95% confidence interval) for the prevalence of cardiovascular risk factors at inclusion in participants with bad work environment according to their past experience of unemployment.

|  | **Past unemployment** | **n** | **%** | **Models 1** | **p** | **Models 2** | **p** |
| --- | --- | --- | --- | --- | --- | --- | --- |
| **Non-moderate**  **alcohol consumption** | **Never** | 4750 | 13.2 | 1.00 |  | 1.00 |  |
|  | **At least once** | 1020 | 14.2 | 1.15 (1.07-1.24) | 0.0002 | 1.07 (0.99-1.16) | 0.08 |
| **Smoking** | **Never** | 6368 | 17.7 | 1.00 |  | 1.00 |  |
|  | **At least once** | 1769 | 24.7 | 1.45 (1.36-1.54) | <0.0001 | 1.24 (1.16-1.33) | <0.0001 |
| **Leisure-time**  **physical inactivity** | **Never** | 2541 | 7.1 | 1.00 |  | 1.00 |  |
|  | **At least once** | 661 | 9.2 | 1.27 (1.16-1.39) | <0.0001 | 1.15 (1.05-1.27) | 0.003 |
| **Obesity** | **Never** | 5081 | 14.1 | 1.00 |  | 1.00 |  |
|  | **At least once** | 1239 | 17.3 | 1.29 (1.21-1.38) | <0.0001 | 1.21 (1.12-1.30) | <0.0001 |
| **Diabetes** | **Never** | 1019 | 2.8 | 1.00 |  | 1.00 |  |
|  | **At least once** | 234 | 3.3 | 1.38 (1.19-1.59) | <0.0001 | 1.27 (1.09-1.48) | 0.002 |
| **Sleep disorders** | **Never** | 23,740 | 65.9 | 1.00 |  | 1.00 |  |
|  | **At least once** | 4998 | 69.6 | 1.15 (1.09-1.21) | <0.0001 | 1.14 (1.08-1.21) | <0.0001 |
| **Depression** | **Never** | 5546 | 15.4 | 1.00 |  | 1.00 |  |
|  | **At least once** | 1693 | 23.6 | 1.57 (1.47-1.67) | <0.0001 | 1.33 (1.24-1.42) | <0.0001 |

The percentages were calculated relatively to the number of participants in each past experience of unemployment (never=36,042; at least once=7176).

Models 1 were adjusted for sex and age.

Models 2 were adjusted for sex, age, current unemployment and social position.
