# Supplementary material for: Cardiovascular burden and unemployment: A retrospective study in a large population-based French cohort
Source: PLoS One. 2023 Jul 17;18(7):e0288747. doi: 10.1371/journal.pone.0288747 (PMC10351739; doi:10.1371/journal.pone.0288747)
Supplement: S13 Table — (DOCX) [file pone.0288747.s016.docx]

**S13 Table:** Adjusted odds ratios (95% confidence interval) for the prevalence of non-fatal myocardial infarction and peripheral arterial disease at inclusion in participants with bad work environment according to their past experience of unemployment.

|  | **Past unemployment** | **n** | **%** | **Models 1** | **p** | **Models 2** | **p** | **Models 3** | **p** |
| --- | --- | --- | --- | --- | --- | --- | --- | --- | --- |
| **Myocardial**  **infraction** | **Never** | 439 | 1.22 | 1.00 |  | 1.00 |  | 1.00 |  |
|  | **At least once** | 98 | 1.37 | 1.42 (1.14-1.78) | 0.002 | 1.42 (1.12-1.79) | 0.003 | 1.31 (1.03-1.67) | 0.03 |
| **Peripheral**  **arterial disease** | **Never** | 144 | 0.40 | 1.00 |  | 1.00 |  | 1.00 |  |
|  | **At least once** | 36 | 0.50 | 1.48 (1.02-2.14) | 0.04 | 1.38 (0.94-2.03) | 0.10 | 1.21 (0.82-1.78) | 0.34 |

The percentages were calculated relatively to the number of participants in each past experience of unemployment (never=36,042; at least once=7176).

Models 1 were adjusted for sex, age and parental history of cardiovascular event.

Models 2 were adjusted for sex, age, parental history of cardiovascular event, current unemployment and social position.

Models 3 were adjusted for sex, age, parental history of cardiovascular event, current unemployment, social position, lifetime alcohol consumption, smoking, leisure-time physical inactivity, obesity, hypertension, dyslipidemia, diabetes, sleep disorders and depression.
