# Supplementary material for: Cardiovascular burden and unemployment: A retrospective study in a large population-based French cohort
Source: PLoS One. 2023 Jul 17;18(7):e0288747. doi: 10.1371/journal.pone.0288747 (PMC10351739; doi:10.1371/journal.pone.0288747)
Supplement: S14 Table — (DOCX) [file pone.0288747.s017.docx]

**S14 Table:** Adjusted odds ratios (95% confidence interval) for the prevalence of cardiovascular risk factors at inclusion in participants with bad work environment according to their current experience of unemployment.

|  | **Current unemployment** | **n** | **%** | **Models 1** | **p** | **Models 2** | **p** |
| --- | --- | --- | --- | --- | --- | --- | --- |
| **Non-moderate**  **alcohol consumption** | **No** | 4892 | 13.1 | 1.00 |  | 1.00 |  |
|  | **Yes** | 878 | 14.9 | 1.35 (1.25-1.47) | <0.0001 | 1.32 (1.21-1.44) | <0.0001 |
| **Smoking** | **No** | 6179 | 16.6 | 1.00 |  | 1.00 |  |
|  | **Yes** | 1958 | 33.1 | 1.58 (1.48-1.69) | <0.0001 | 1.43 (1.34-1.53) | <0.0001 |
| **Leisure-time**  **physical inactivity** | **No** | 2526 | 6.8 | 1.00 |  | 1.00 |  |
|  | **Yes** | 676 | 11.4 | 1.29 (1.17-1.41) | <0.0001 | 1.19 (1.07-1.31) | 0.0007 |
| **Obesity** | **No** | 5496 | 14.7 | 1.00 |  | 1.00 |  |
|  | **Yes** | 824 | 13.9 | 1.13 (1.04-1.23) | 0.005 | 1.02 (0.93-1.11) | 0.69 |
| **Depression** | **No** | 5596 | 15.0 | 1.00 |  | 1.00 |  |
|  | **Yes** | 1643 | 27.8 | 1.63 (1.52-1.74) | <0.0001 | 1.43 (1.33-1.54) | <0.0001 |

The percentages were calculated relatively to the number of participants in each current experience of unemployment (no=37,306; yes=5912).

Models 1 were adjusted for sex and age.

Models 2 were adjusted for sex, age, past unemployment and social position.
